# A Large Accumulation of Avian Eggs from the Late Cretaceous of Patagonia (Argentina) Reveals a Novel Nesting Strategy in Mesozoic Birds

**DOI:** 10.1371/journal.pone.0061030

**Published:** 2013-04-17

**Authors:** Mariela S. Fernández, Rodolfo A. García, Lucas Fiorelli, Alejandro Scolaro, Rodrigo B. Salvador, Carlos N. Cotaro, Gary W. Kaiser, Gareth J. Dyke

**Affiliations:** 1 Instituto de Investigaciones en Biodiversidad y Medioambiente, INIBIOMA - CONICET, San Carlos de Bariloche, Río Negro, Argentina; 2 Instituto de Investigación de Paleontología y Geología, Museo “Carlos Ameghino”, Universidad Nacional de Río Negro, Cipolletti, Río Negro, Argentina; 3 Centro Regional de Investigaciones Científicas y Transferencia Tecnológica, CRILAR-CONICET, Anillaco, La Rioja, Argentina; 4 Cátedra de Ecología, Universidad Nacional de la Patagonia San Juan Bosco y CENPAT-CONICET, Puerto Madryn, Chubut, Argentina; 5 Museu de Zoologia, Universidade de São Paulo, São Paulo, São Paulo, Brazil; 6 Caracterización de Materiales, Centro Atómico Bariloche, San Carlos de Bariloche, Río Negro, Argentina; 7 Natural History, Royal British Columbia Museum, Victoria, British Columbia, Canada; 8 Ocean and Earth Science, National Oceanography Centre, University of Southampton, Southampton, United Kingdom; Raymond M. Alf Museum of Paleontology, United States of America

## Abstract

We report the first evidence for a nesting colony of Mesozoic birds on Gondwana: a fossil accumulation in Late Cretaceous rocks mapped and collected from within the campus of the National University of Comahue, Neuquén City, Patagonia (Argentina). Here, Cretaceous ornithothoracine birds, almost certainly Enanthiornithes, nested in an arid, shallow basinal environment among sand dunes close to an ephemeral water-course. We mapped and collected 65 complete, near-complete, and broken eggs across an area of more than 55 m^2^. These eggs were laid either singly, or occasionally in pairs, onto a sandy substrate. All eggs were found apparently in, or close to, their original nest site; they all occur within the same bedding plane and may represent the product of a single nesting season or a short series of nesting attempts. Although there is no evidence for nesting structures, all but one of the Comahue eggs were half-buried upright in the sand with their pointed end downwards, a position that would have exposed the pole containing the air cell and precluded egg turning. This egg position is not seen in living birds, with the exception of the basal galliform megapodes who place their eggs within mounds of vegetation or burrows. This accumulation reveals a novel nesting behaviour in Mesozoic Aves that was perhaps shared with the non-avian and phylogenetically more basal troodontid theropods.

## Introduction

**Figure 1:**
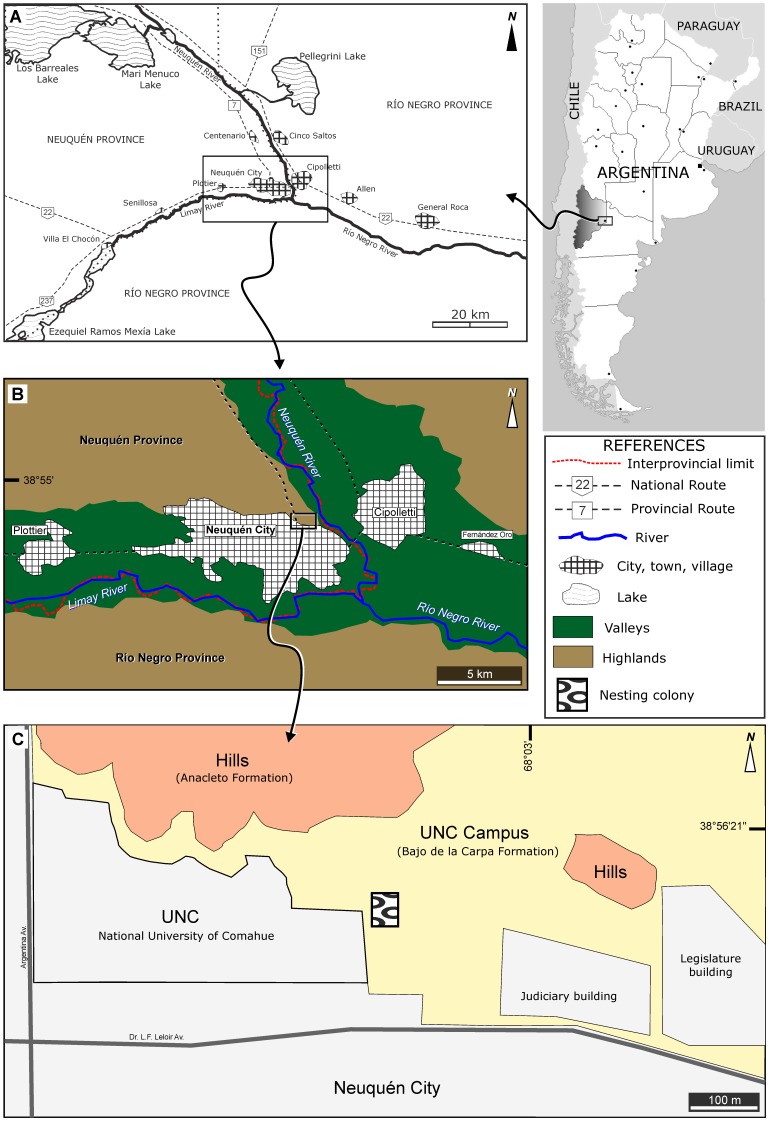
Site location. Counter-clockwise from top right: map of Argentina and the Comahue region (Neuquén and Río Negro Provinces); map of Neuquén Province; map of Neuquén City; close up of Neuquén City to show the University of Comahue (UNC) campus, and the location of the fossil bird nesting colony (shaded box).

In the 1980s a team from the National University of Comahue (Patagonia: Argentina) collected a large number of eggshell fragments along with some intact whole eggs from the late Cretaceous Bajo de la Carpa Formation in Neuquén City, Patagonia (Figure 1). Part of this fossil collection was later described by Schweitzer et al. [1] who reported that some of the eggs contained embryonic bone fragments (MUCPv 284, 305, 306) and one an articulated embryo (MUCPv 284). Schweitzer et al. [1] assigned these fossil remains to basal birds, showing, on the basis of preserved embryonic anatomy, that they were certainly ornithothoracines, and most likely enantiornithines. This report [1] was the first to associate the anatomy of a Cretaceous bird with preserved eggshell morphology and was soon followed by others [2], [3]. Later, Grellet-Tinner et al. [4] studied eggs from this collection and interpreted the loss of the polar and immediately adjacent regions as evidence of hatching and thereby a specific hatching strategy typical of modern birds. Most recently, Dyke et al. [5] described a fossil association of jumbled eggshell, adult and juvenile bones and complete eggs (lacking embryonic remains) from the Late Cretaceous of Transylvania (Romania) that they interpreted as the remains of a nesting colony.

Here, we significantly augment the known fossil record of Cretaceous birds by presenting the first known concentration of contemporaneous and complete avian eggs preserved in their laid positions. Unlike the jumbled and broken accumulation reported by Dyke et al. [5], the positions of these Argentine fossil eggs allow collection of spatial information bearing on nesting and hatching behaviour. Further, many of these eggs contain isolated broken bones, some partially ossified. Estimates of shell water vapour conductance (G_H2O_) enable us to establish palaeoecological context and infer the likely palaeobiology of this Argentine Cretaceous bird breeding colony.

## Materials and Methods

### Institutional Abbreviations

CRILAR, Centro Regional de Investigaciones Científicas y Transferencia Tecnológica La Rioja, La Rioja Province, Argentina; MUCP, Museo de Geología y Paleontología, Universidad Nacional del Comahue, Neuquén, Argentina.

### *In Situ* Eggs and Eggshells

We mapped 65 eggs within the campus of the Universidad Nacional del Comahue, North of Neuquén city, Argentina (Figure 1) and obtained permission from the MUCP to access their collections and to research this fossil material. No permits were required for this research. Among the eggs we collected, many contain embryonic remains (MUCPv 1354 to 1358) while some are almost complete but lack bony remains (MUCPv 37, 189, 235, 238, 283/1, 283/2, 285, 286, 307, 1239, 1240). Other eggs are partially preserved (MUCPv 36, 236/1, 236/2, 237/1, 237/2, 260, 1241–1249, 1251–1257, 1271) and some are just large shell pieces (MUCPv 1258–1270, 1272, 1359–1366). In addition, some eggs lack complete shells so are preserved only as endocasts (MUCPv 235, 286). We made thin section preparations of MUCPv 1258, 1259 and 1260 and used SEM to reveal the ultrastructure in some broken egg pieces and complete eggs (MUCPv 1258, 1259 and 1260). Schweitzer et al. [1] studied MUCPv 305, 306, 350–355 and the embryo MUCPv 284.

For comparisons, we also include one well-preserved and two poorly preserved eggs collected in 2006 from the eastern Comahue campus and loaned to one of us (LEF) by Dr. L. Salgado. These specimens are housed in the CRILAR collections (CRILAR-Pv 410a, 410b and 410c).

**Figure 2:**
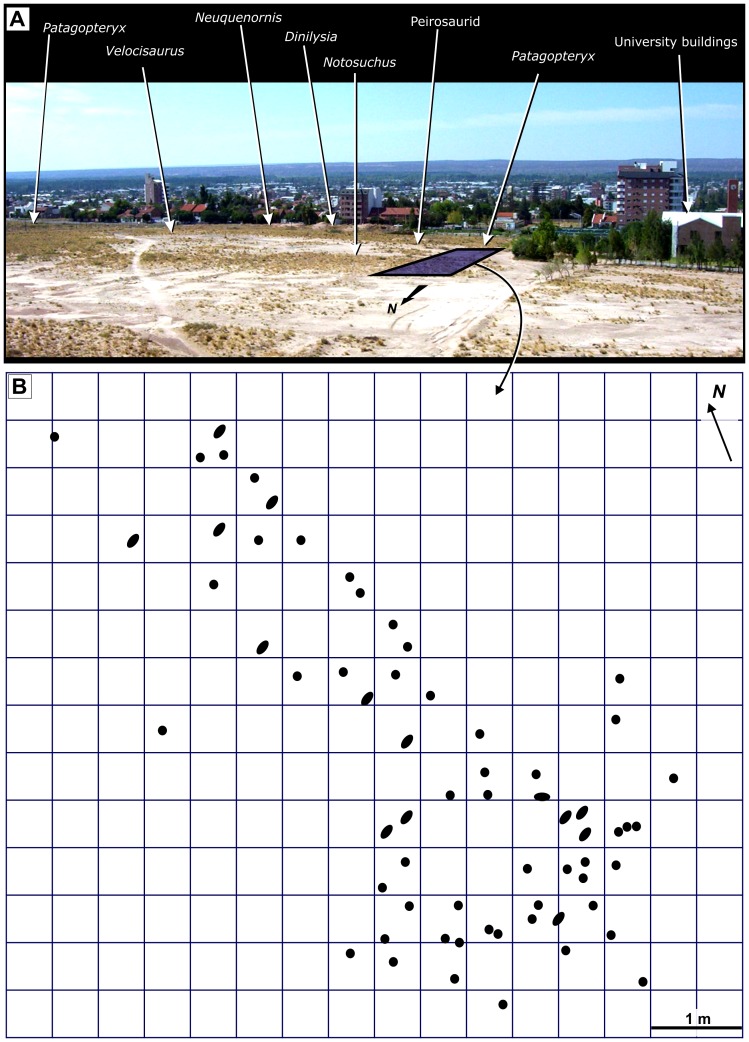
Close-up of site location and *in situ* egg map. Top: overview of the Universitary campus showing the location of several paleofaunal elements and the grid corresponding to the nesting colony (shaded purple box). Bottom: grid showing the location of each mapped egg; circles represent upright eggs, ovals represent eggs slightly inclined vertically and the oval that lies with its long axis parallel to the substrate represents an egg found in that position.

To map this fossil accumulation we placed a 0.25-m^2^ grid over an area of 45.25 m^2^ (Figure 2), an accurate approach because bedding in this area is horizontal with little or no dip (see Geological Setting). Eggs present in each grid square were counted and their distribution analyzed using a χ^2^test in SigmaStar 3.5. modelling the eggs using a Poisson distribution. We were able to determine whether their distribution on the ground was random, continuous or uniform [6] and calculated χ^2^ for two degrees of freedom. We examined the microscopic details of the shells using SEM and prepared thin sections of eggshell using standard methods [7].

To calculate egg volume we made a silicone mold of one complete egg (MUCPv 1240) and calculated its displaced volume. We then verified this estimate mathematically using the volumetric formula for ellipsoids: V = 4/3 π.a.b.c (a: length/2;b: width/2; c: width/2).

### Water Vapour Conductance

The structural and functional properties of eggshell are paramount determinants of the incubation and hatching success of reptile and bird embryos [8]. One of the main physiological properties of an egg is shell permeability, or conductance to both respiratory gases and water vapour. Gas diffusion through the eggshell pores can be quantified as water vapour conductance (G_H2O_). This measure is commonly obtained experimentally for modern bird and reptile eggs [9], but has rarely been estimated for fossils. From fossil eggs, however, G_H2O_ can be determined by simple equations and thus represents a valuable proxy for assessing moisture content in archosaurian nesting environments and potentially can provide additional information on parental nesting strategies [10], [11]. G_H2O_ estimates for a number of dinosaur eggs have been published [11]–[13] but this parameter has never been computed for a Cretaceous fossil bird egg other than the enantiornithine *Gobipteryx* [11], [14].

In order to obtain G_H2O_ estimates for the Comahue fossil eggs, we used a well-established equation for extant birds [15]. Two oological parameters are required for this calculation, egg density and egg radius (Table 1), and we approximated the shape of the fossil eggs as ellipsoids (prolate spheroids). Thus, there are two radii: Equatorial radius (α) and polar radius (β). Egg density was inferred from extant bird eggs following Paganelli et al. [16]; for comparative purposes we have also tabulated predicted G_H2O_ values for bird and non-avian theropod eggs from other published sources, including the Mongolian *Gobipteryx minuta* (Table 2). Note that these G_H2O_ estimates were calculated from eggshell thin sections, not the equation-based approach we use here (see Discussion).

**Table 1:** Summary of all parameters, units, equations and results for the Comahue eggs (N = 65).

Parameters	Unit	Formula/Method	*N. volans*
α Equatorial radius	cm	data from observation	1.35
β Polar diameter	cm	data from observation	2.25
ε Angular eccentricity of elipse	-	ε = arccos (α/β)	0.93
As Egg surface area	cm^2^	As = 2 π+[α^2^+(αβε/sin (ε))]	33.57
V Egg volume	Cm^3^	V = (4/3)·π α^2^ β	17.18
ρ Egg density	g/cm^3^	Assumed from avian egg data	1.08
m Egg mass	g	m = ρ·V	19.96
G_H2O_ Water vapor conductance	mg_H2O_/day·Torr	G_H2O_ = 0.384·m^0.814^	4.39

Calculations in this table use formulae from [15].

**Table 2:** Predicted G_H2O_ values (in mgH20/day·Torr) for the Comahue eggs (based on Table 1 and formulae in [15]) alongside those for other taxa from previous studies (i.e., G_H2O_ values estimated using pore counts from egg shell thin sections).

Source	Locality	Egg	G_H2O_
**This study**	Neuquén, Argentina	*Neuquenornis volans* (enantiornithine bird)	4.39
**Sabath (1991)**	Gobi Desert, Mongolia	*Gobipteryx minuta* (enantiornithine bird)	2.7
**Deeming (2006)**	Portugal	*Lourinhanosaurus antunesi* (theropod)	541
**Deeming (2006)**	China	*Macroelognatoolithus xixianensis* ( theropod)	600
**Deeming (2006)**	Canada	*Prismatoolithus levis* (troodontid theropod)	39
**Deeming (2006)**	Gobi Desert, Mongolia	*Gobipteryx minuta* (enantiornithine bird)	2.5
**Ar et al. (1974)**	Africa	*Struthio camelus* (ostrich)	105
**Ar et al. (1974)**	Australia	*Dromiceius novaehollandiae* (emu)	51.8
**Ar et al. (1974)**	Holartic	*Larus argentatus* (herring gull)	16.5
**Ar et al. (1974)**	Cosmopolitan	*Gallus gallus* (domestic chiken)	14.4
**Ar et al. (1974)**	Asia	*Phasianus colchicus* (ring-necked phaesant)	6.6
**Ar et al. (1974)**	East Asia	*Coturnix coturnix* (Japanese quail)	3.1
**Ar et al. (1974)**	North America	*Quisculus quiscula* (common grackle)	2.3
**Ar et al. (1974)**	Cosmopolitan	*Passer domesticus* (house sparrow)	0.9

Note that: (1) because different approaches were used to predict G_H2O_ values (regressions versus pore counts from thin sections) they may not be comparable; and (2) value shown for *G. minuta* is that predicted when compared to an avian egg of similar size, not G_H2O_, which has been estimated to range from 63.9 [11] to 22.4 [14]. See [11] for further discussion.

### Geological Setting

**Figure 3:**
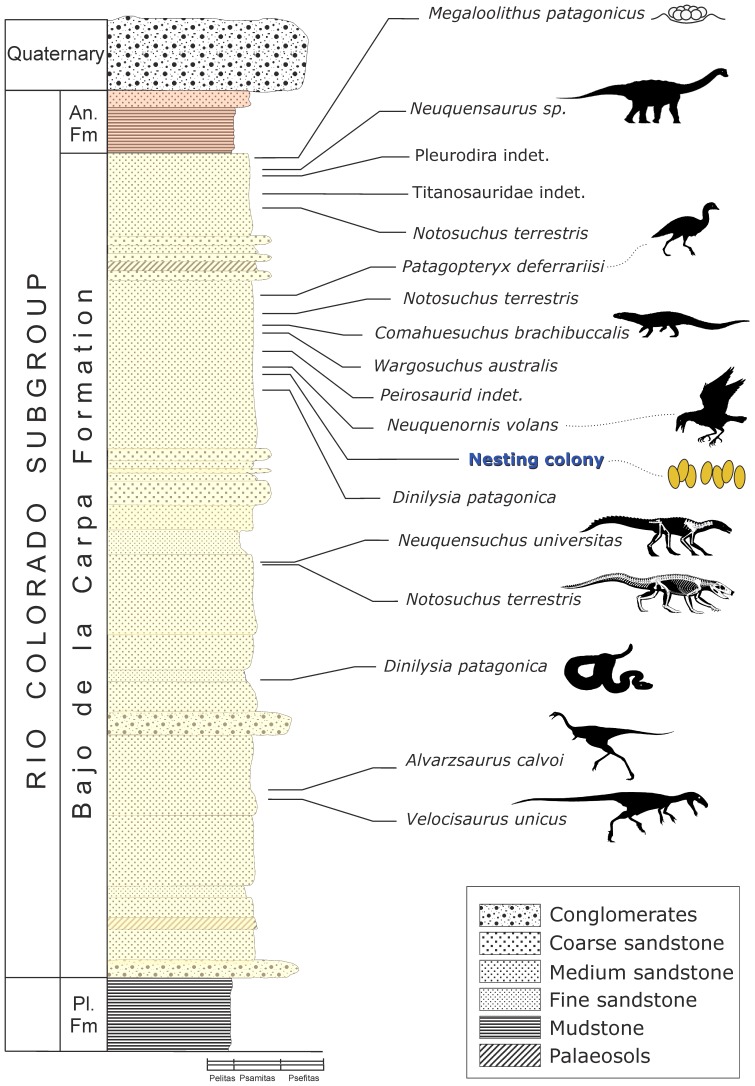
Stratigraphic profile. Bajo de la Carpa Formation, Neuquén Group [17], [31] to illustrate the diverse biota spread through the horizons within this sequence. Layer containing the eggs discussed in this paper is indicated. Abbreviations: An, Anacleto Formation; Pl, Plottier Formation.

This accumulation of fossil bird eggs was found on exposed beds that have been referred to the Bajo de la Carpa Formation (Río Colorado Subgroup, Neuquén Group; Middle-Upper Santonian) [17]–[19] (Figure 3). The Bajo de la Carpa Formation rests conformably on the Plottier Formation and is capped by deposits of the Anacleto Formation (Figure 3) [19]. Unlike other regions of the Neuquén Basin, the Santonian rocks of this formation were deposited by fluvial and aeolian systems [20] as well as extensive flood plains [18], [19] (Figure 3).

The yellow quartz-rich sandstone of the Bajo de la Carpa Formation contains poorly sorted, subangular-to-subrounded grains of low sphericity; these generally monocrystalline quartz grains range between 0.1 to 0.5 mm in diameter, producing a fine-to-medium sandstone that does not contain feldspars, mica fragments or any associated lithics (see [19], [20]). It has a clay matrix and calcareous cement – a microspar– that is ferric and light in colour. The isopachous carbonate cement is secondary (diagenetic) [19], [20], formed in a waterlogged environment [18].

Regionally, this system formed in an arid and dry continental climate via aeolian deposition [18], [19]; there is clear variation from fluvial systems to distal floodplains across the sequence with increasing participation of aeolian sediments [19]. The palaeoeonviroment inferred for the university campus area consists of aeolian deposition that created large dunes and inter-dune lagoon basins skirted by fluvial deposits, criss-crossed by streams and seasonal or ephemeral water bodies [18], [21].

Aeolian deposits offer little resistance to the movement of groundwater. As a result they are subject to sudden changes in the water table when distant rains raise the level of local streams or raise the level of the local ground water. In a relatively flat area, such as Comahue, relatively small quantities of water at the surface could cover a large area.

### Palaeontological Context

The Comahue eggs come from the same stratigraphic level as some parts of a rich, associated paleofauna (fossils collected from beds throughout the Bajo de la Carpa Formation), dominated by crocodyliforms [18], [22], [23] (Figures 2, 3). Indeed, the Bajo de la Carpa crocodilian fauna is well-known and includes the plesiomorphic crocodyliform *Neuquensuchus universitas* [24], [25], notosuchians *Notosuchus terrestris* [26] and *Comahuesuchus brachybuccalis* [22], baurusuchids *Cynodontosuchus rothi* [26] and *Wargosuchus australis* [27], as well as a peirosaurid crocodyliform of uncertain phylogenetic position [18]. The remainder of the fauna includes skeletal material referred to the basal snake *Dinilysia patagonica* [28], [29], the abelisauroid theropod *Velocisaurus unicus* [22], [30], the alvarezsaurid theropod *Alvarezsaurus calvoi* [22], and the enantiornithine and basal ornithuromorph birds *Neuquenornis volans* [31] and *Patagopteryx deferrariisi* [32] (Figure 2).

## Results

### Egg Morphology and Contents

**Figure 4:**
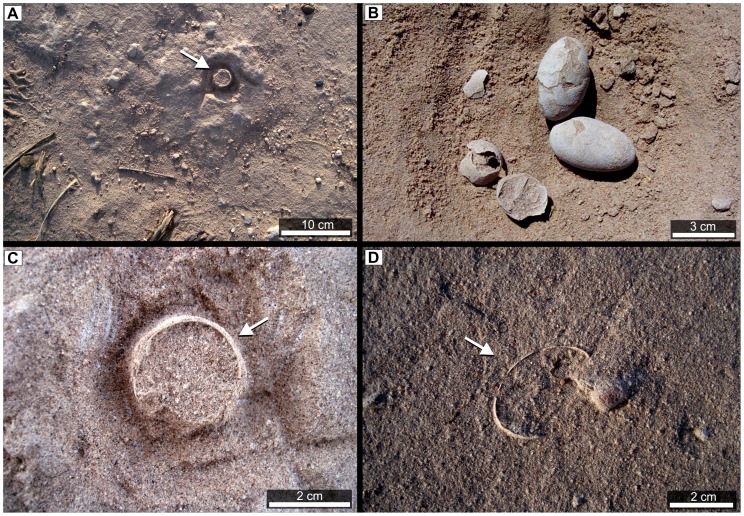
Several *in-situ* complete and fragmentary eggs from the Comahue campus nesting colony. These eggs demonstrate spatial arrangement in vertical (*a* and *c*), subvertical (*d*) and horizontal (*b*) positions.

Although a small number of the fossil eggs from the Comahue campus have been reported before [1], [4], previous work focused on isolated specimens examined outside the context of this remarkable accumulation of *in situ* eggs (Figures 2, 4). Indeed, based on preserved microstructure and embryonic anatomy, we concur with previous workers that the Comahue eggs were laid by ornithothoracine birds. Embryonic bones inside the eggs include strut-like coracoids and wide ulnae associated with small, narrow radii [1], synapomorphies of Ornithothoraces [33]–[35]. As noted above, adult fossil remains of two ornithothoracine birds, the enantiornithine *Neuquenornis* [31] and the more basal *Patagopteryx* [32] are known from the same area.

Many of the partial eggs in the collection (n = 22) appear to lack their larger pole, suggesting that they had hatched prior to any flooding event [4]. However, the peculiar vertical posture of these eggs (Figure 4) would also have exposed the large ends to a greater risk of damage from accident or small predators and scavengers.

**Figure 5:**
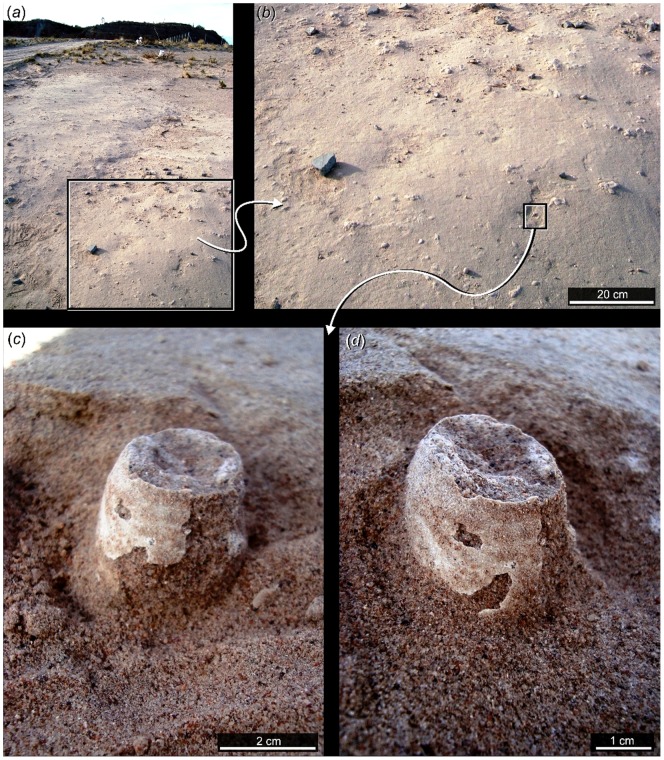
*In situ* eggs within the Comahue campus. *In situ* association in bedding plane (*a*), inset of single egg in position (*b*), egg half-buried in sediment (typical for almost all eggs collected) (*c*), close up lateral view of same egg showing degree of asymmetry (*d*).

**Figure 6:**
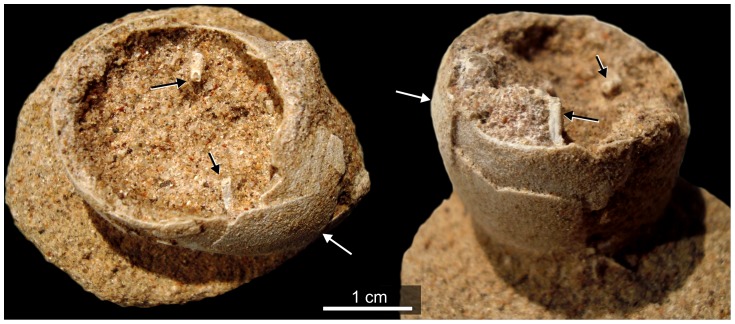
Partial egg (MUC-Pv 1358) with typically fractured but well preserved eggshell. Some avian bone fragments are visible inside the egg (arrows).

We mapped and collected all but one of the most complete eggs from this site. These specimens (n = 46) are between 41 and 47 mm (±0.01) in length and have equatorial diameters that range between 26 and 29 mm (Figures 4, 5, 6). External eggshell surfaces are smooth and cream-coloured and complete eggs are ellipsoid with only one axis of symmetry (Figures 4, 5, 6). Like the eggs of most modern birds the specimens lack surface ornamentation (as noted previously by Schweitzer et al. [1] and Grellet-Tinner et al. [4] (Figure 6). Many exhibit diagenetic surface textures as a result of abrasion, corrosion and dissolution. Average shell thickness is 180 µm (±2,5 µm); volume was estimated for complete eggs MUCPv 1240, MUC-Pv 307 and CRILAR-Pv 410a as 19.5, 18.77, and 17.18 cm^3^, respectively. These estimates are similar to data for living plovers (e.g. *Vanellus chilensis* (18.37 cm^3^), *Arenaria interpes* (19.36 cm^3^) and other Charadriiformes [36], [37]. There is only a weak correlation between egg size and body size. *V. chilensis* is slightly larger (35–37 cm total body length) but very much heavier (327 g) than *A. interpes* (21–25 cm and 136 g) [36]–[38]. Nonetheless, the egg volumes suggest an association between the Comahue eggs and the enantiornithine *Neuquenornis* in agreement with Schweitzer et al. [1]. This enantiornithine was much smaller than examples of *Patagopteryx* from the same locality [32], [33].

### Spatial Distribution of Eggs

We mapped 65 complete and partial eggs *in situ* (Figure 2). Almost all are separated by just over their own length from their neighbour as is typical of exceptionally dense avian colonies [39]. We found only a single pair of eggs and one group of three (Figure 2). All eggs occur in a broad band that is oriented north-south and that certainly extended further to the north than we are now able to explore (Figure 2).

Once mapped in two dimensions (Figure 2), all eggs (apart from one) were collected. The majority were buried vertically in the sediment with their polar region pointing downwards (Figure 4a–c); only in a few rare cases were eggs found lying horizontally on the ground (Figure 4d), presumably because of disturbance after burial. A chi-squared test (χ^2^ = 348; N = 182; P<0.001) shows that this egg concentration (Figure 2) is non-random in distribution and strongly suggests that birds congregated in this area to breed. Our interpretation of a nesting colony is supported by the fact that the embryonic remains identified inside several eggs are all in a similar state of fairly advanced development, have hatched [4] or are broken and lack bones completely. This mixture of hatched and developing eggs is a characteristic of modern avian nesting colonies [39]–[41].

### Water Vapour Conductance

We calculated an average G_H2O_ of 4.14 mg_H2O_/day·Torr for the Comahue eggs (Table 1) which lies well within the lower end of the known distribution for modern bird eggs [9] (Table 2). Such a low G_H2O_ value indicates relatively little water vapour loss from the Comahue eggs and implies an ability to use a dry nesting area with low relative humidity, such as the paleoenvironment inferred for the Bajo de la Carpa Formation [18]–[20].

## Discussion

Descriptions and our map of the Comahue eggs (Figure 2) strongly support interpretation of this fossil accumulation as the remains of a Cretaceous bird nesting colony. The *in situ* preservation of eggs, in combination with well-preserved surface textures (Figures 4, 5, 6) demonstrate minimal (if any) taphonomic disturbance prior to burial [42]. Field observations are consistent with a high degree of synchronicity [42], typical of other amniote egg and nest fossils interpreted as representing colonies from the Cretaceous of Romania [5], Asia (Gobi Desert) [14] and from the Sanagasta neosauropod nesting site in La Rioja, Argentina [43]. Egg and nest associations will be quickly disturbed and disarticulated by post-burial processes, even in inferred low energy environments (enantiornithine eggs and nests, for example, from Romania [5]). Low degrees of taphonomic disturbance are also characteristic of the other fossil vertebrates from the Universitary Campus area [18]. Indeed, we argue that the fossils from this area comprise a “census assemblage” (Model I) (*sensu* [18]): A very high proportion of articulated remains often with surfaces in pristine condition and some –if not most–in their positions at the moment of death [44], [45]. Moreover, because the concentration of the Comahue eggs is “intrinsic” (*sensu* [46]), this association could only have been produced by the gregarious behaviour of colonial organisms [45].

The ellipsoidal shape of the eggs at Comahue is typical of modern eggs laid in a clutches of 3 to 8 and may be related to incubation efficiency [47]. Except in special situations such as the placement of nest sites on cliff edges, nearly spherical eggs are the most efficient shape for single-egg clutches. In this case, the vertical posture allows the egg to comply with the prediction of Barta and Székely [47] by exposing a spherical surface to the incubating adult.

If the partial eggs at Comahue share internal morphology with modern birds, their missing poles would have held the air cell [48] while the disappearance of the shell from that part of the partial eggs implies that enantionithine birds had already adopted a hatching behaviour favoured by modern neornithine birds [4]. However, the vertical placement of solitary eggs in an open nest is unknown among modern birds. Only the megapode, a basal galliform, deposits its eggs vertically but then only in clutches buried in a heap of decomposing vegetation, tree roots or burrows. Its eggs are not asymmetrical and numerous small air cells are scattered around the embryo [49]. The Comahue eggs resemble those of some troodontids that placed their eggs vertically and appear to have hatched by breaking out through the upper pole [50]. The north-south linear arrangement of this accumulation is also significant; such linearity is often characteristic of extant bird colonies established along the edge of a stream or cliff [39], [41].

Among extant neornithine birds, the most similar nesting strategy is use of a simple “scrape” [49]. A scrape is typically just a shallow depression with enough of a rim to keep eggs from rolling away [49]. At Comahue, a nest structures appears to be just sufficient to prevent the eggs toppling over. Use of these simple scrapes is seen in several paleognaths [51] and many neognaths, including members of the Galloanserinae, Charadriiformes, Falconiformes, Caprimulgiformes, Otidae, and Pteroclidae [37]. Some charadriiforms, in particular terns (Sternidae), breed in colonies broadly comparable to the Comahue accumulation, often on a sandbar or a beach where their scrapes are situated on barren or sparsely vegetated areas near water [52].

Our low prediction for the water vapour conductance (G_H2O_) of the Comahue eggs (Table 1) is consistent with geological observations. An arid and dry environment for the site, also inferred by calculated G_H2O_ values, is corroborated by the local sedimentology [18], [20]. Further, the absence of any nesting structures and the fact that the Comahue eggs were all half-buried *in situ* suggests that the upper portion of these eggs were exposed on the surface after laying and would thus require an attending brooding parent [11]. A similar strategy has been implied for the enantiornithine *Gobypterix minuta* [53] for which we predict an even lower G_H2O_ value, 2.5–2.7 mg_H2O_/day·Torr (Table 2).

Corresponding with observations on living birds [11], [15], these relatively low values of G_H2O_ may have allowed the Comahue birds to exploit drier patches of habitat. Indeed, generally much higher G_H2O_ values that have been predicted for the phylogenetically more basal non-avian theropods [11] (Table 2) (egg size notwithstanding) are an order of magnitude greater than for any living birds and may suggest that these taxa required more humid nesting environments or had more elaborate nests [11], [13]. Comparisons of similar-sized eggs (once found and collected from the fossil record) between birds and non-avian theropods will, however, be required to corroborate our speculation. Nevertheless, predicted G_H2O_ values are significantly lower for some small, non-avian theropods that are considered phylogenetically close to birds, including troodontids [11], [54]. These fall well within the extant avian range yet are still higher than those predicted for Cretaceous fossil birds (Table 2): low values are consistent with the suggestion that nests of the North American *Troodon formosus* were attended by a brooding parent [54]. Parental care has also already been well-established in the closely-related oviraptorid *Oviraptor philoceratops* [55].

We conclude that the Comahue fossil bird eggs present an interesting mixture of primitive and advanced traits. On the one hand, it appears likely that embryos were ventilated by a single, large air chamber and used a strategy considered distinctively avian for exiting the egg [1], [4] (although this has also been proposed to have been the case for troodontids [11], [54]), while on the other eggs were laid vertically and could not have been turned by the parent. Egg turning is widespread in extant birds and has been intensively studied in galliforms for the poultry industry [56]. This behaviour is believed to place the embryo in an opportune position and allow effective functioning of the connections between the embryo and the yolk sac: the unturned eggs of domestic fowl have a much higher mortality rate (85 percent) and take seven hours longer to hatch [56]. The incubation period for vertically-placed megapode eggs (that are buried and cannot be turned) is four or five times that of domestic fowl [57]. We speculate that the vertical nesting strategy evidenced by the Comahue eggs was abandoned by later lineages because it was not competitive with the greater incubation success and reduced incubation time of turned eggs. Among modern birds, even basal-most lineages (including some palaeognaths) contain at least some species that turn their eggs [58], [59], [60].
